# Typologies of interactions between abortion seekers and healthcare workers in Australia: a qualitative study exploring the impact of stigma on quality of care

**DOI:** 10.1186/s12884-023-05902-0

**Published:** 2023-09-07

**Authors:** Shelly Makleff, Madeleine Belfrage, Sethini Wickramasinghe, Jane Fisher, Deborah Bateson, Kirsten I. Black

**Affiliations:** 1 Global and Women’s Health, School of Public Health and Preventive Medicine, Monash University, Level 4, 553 St Kilda Rd, Melbourne, VIC 3004 Australia; 2 School of Social Science, The University of Queensland, Forgan Smith Building, St Lucia, Brisbane, QLD 4072 Australia; 3 Faculty of Medicine and Health, The University of Sydney, Science Road, Camperdown, Sydney, NSW 2050 Australia

**Keywords:** Abortion, Stigma, Quality of care, Lived experience, Qualitative research, Australia, Structural stigma

## Abstract

**Background:**

Abortion stigma involves the stereotyping of, discrimination against, and delegitimization of those who seek and provide abortion. Experiences of abortion care are shaped by stigma at the meso (e.g., lack of local providers) and macro (e.g., abortion regulations) levels. Yet abortion stigma and quality of care are often examined separately. This study sought to articulate the impact of abortion stigma on quality of care in the context of healthcare interactions. It did so by characterizing the features of stigmatizing and non-stigmatizing care in the context of macro-level stigma and other structural factors that influence abortion-seeking experiences, including the coronavirus pandemic’s influence on the health system.

**Methods:**

This qualitative study comprised in-depth interviews with people who sought abortion across Australia between March 2020 and November 2022, recruited through social media and flyers in clinics. Thematic analysis drew on concepts of micro, meso, and macro stigma and person-centered care. We developed typologies of the interactions between abortion seekers and healthcare workers by analytically grouping together negative and positive experiences to characterize features of stigmatizing and and non-stigmatizing care in the context of macro-level influences.

**Results:**

We interviewed 24 abortion seekers and developed five typologies of stigmatizing care: creating barriers; judging; ignoring emotional and information needs; making assumptions; and minimizing interactions. There are five corresponding positive typologies. Macro-level factors, from abortion regulations to rural and pandemic-related health system pressures, contributed to poor experiences in care.

**Conclusions:**

The positive experiences in this study illustrate how a lack of stigma enables patient-centered care. The negative experiences reflect the interrelationship between stigmatizing beliefs among healthcare workers, macro-level (policy and regulatory) abortion stigma, and structural health service limitations exacerbated during the pandemic. Interventions are needed to reduce stigmatizing interactions between abortion seekers and healthcare workers, and should also consider macro-level factors that influence the behaviors of healthcare workers and experiences of abortion seekers. Without addressing stigma at multiple levels, equitable access to high-quality abortion care will be difficult to achieve. Efforts to integrate stigma reduction into quality improvement have relevance for maternal and reproductive health services globally.

## Background

Stigma is “the co-occurrence of labeling, stereotyping, separation, status loss, and discrimination in a context in which power is exercised” [1]. It is a well-documented barrier to the delivery of high-quality, equitable care for a range of health conditions and services, with health, economic, social, and psychological harms [1, 2]. The elimination of stigma and discrimination in healthcare can be described as “a public health imperative” in line with human rights commitments and initiatives [3].

Abortion is a common and necessary part of reproductive healthcare, with an estimated 61% of unintended pregnancies globally ending in abortion, translating to 73.3 million abortions per year [4]. Despite this, abortion is stigmatized across the globe, reflecting unequal power relations [5] and gendered social norms that control female sexuality and bodies and idealize motherhood [6].

Abortion stigma has been defined as “a shared understanding that abortion is morally wrong and/or socially unacceptable” [7]. It discredits and delegitimizes abortion healthcare and those who seek and provide it, and in doing so limits access to abortion services [8, 9]. Across contexts, abortion seekers lack trusted information sources about abortions and keep their decision a secret to limit judgement or rejection [8–10]. Healthcare providers too are often stigmatized or marginalized for their involvement in abortion care [8, 11].

Rather than conceptualizing abortion stigma “as a set of values, beliefs and judgements that flow from stigmatizers to the stigmatized” at solely an individual level, we understand it as a social process that reproduces unequal power relations [5]. Stigma is increasingly described as intersectional – interacting with and reinforcing other systems of oppression, such as racism, ablism, and sexual orientation and gender discrimination, which also create barriers to healthcare access [12–14]. Abortion stigma operates at interconnected levels: individual, community, institutional, law and policy, and media [6, 7], which can be organized into three categories: micro, consisting of individual experiences and interactions with peers and family; meso, comprising community norms, community groups, and local healthcare providers and facilities; and macro, including legal frameworks and economic, political, and health systems [13]. Individual (micro) experiences of abortion are shaped by abortion stigma at the meso (e.g. clinic/hospital policies restricting abortion provision, refusal of local healthcare staff to provide, or community opposition) and macro (e.g. conscientious objection laws or policies limiting access beyond defined gestational age limits) levels [15].

In the context of abortion seeking, stigma can be enacted in interactions with providers, pharmacists, or other personnel encountered on pathways to abortion care such as medical receptionists and security or cleaning staff (hereafter referred to collectively as ‘healthcare workers’). Enacted abortion stigma can be defined as ‘actual discriminatory behaviors or negative interactions related to abortion experience’ [7]. Abortion seekers around the world commonly anticipate judgement or mistreatment in the health system, and healthcare workers sometimes mistreat abortion seekers or delay their access to services [9, 10, 16–18]. Interactions with healthcare providers are among the most important factors influencing quality of care from the perspective of abortion seekers [19–21], as in maternity [22, 23] and HIV care [24]. Interpersonal interactions on the pathway to abortion care are at the intersection of stigma and quality of care and should be better theorized and understood.

While abortion stigma and abortion quality of care are often examined separately, they are interrelated. A 2022 scoping review by Sorhaindo and Lavelanet examined 50 studies, concluding that stigma is “a significant moderator of quality in abortion care” and identifying seven main ways in which abortion stigma inhibits quality of care [9]. These are poor treatment of abortion seekers; obstruction of access to care; secrecy on the part of abortion providers and seekers; unnecessary and arduous requirements for those seeking and providing care; poor infrastructure and limited resources; punishment of and threats towards both abortion seekers and providers; and a lack of designated spaces for abortion services to be provided. Sorhaindo and Lavelanet’s review [9] drew on previous efforts by the International Network for the Reduction of Abortion Discrimination and Stigma (Inroads) to integrate an abortion stigma lens to the World Health Organization (WHO) quality of care framework [25]. By interviewing abortion seekers in the permissive legal context of Australia [26], our study seeks to build on these emerging efforts by theorizing the link between abortion quality of care and stigma.

The aim of this study was to better articulate the impact of abortion stigma on quality of care in the context of healthcare interactions. We addressed this aim by [1] developing typologies of the interactions between abortion seekers and healthcare workers to characterize features of stigmatizing and non-stigmatizing care, and [2] identifying the influence of macro-level abortion stigma and other structural factors influencing the health system, including the coronavirus pandemic, on interactions and experiences during abortion seeking.

## Methods

### Study setting

Between 2002 and 2021, all states and territories in Australia fully or partially decriminalized abortion [26]. Medication abortion is available until 63 days gestational age [27], while surgical abortion without restriction is available until 14 to 24 weeks, varying by state and territory [26]. Beyond this limit, some jurisdictions require approval of additional providers [26, 28–30]. Accessibility of abortion in Australia varies by location, social circumstances, and economic resources, with amplified barriers in rural and regional areas, among migrant and refugee communities, and for those not covered by national universal healthcare (Medicare) [31, 32]. There is an overreliance on privatized abortion care, with the service mainly provided outside the public system [26]. Abortion access is additionally shaped by structural factors hindering the functioning of the Australian health system, such as the limited capacity of rural health services and, more recently, the COVID-19 pandemic [15, 33]. Despite these barriers, abortion is a common and safe healthcare service in Australia [26, 34, 35]. Drawing on a patchwork of data sources that potentially include dilation and curettage procedures for miscarriage or other gynecological conditions [36], the most recent estimate finds 17.3 abortion procedures per 1000 women of reproductive age annually [36]. This equates to 88,287 abortions per year, or roughly one in three women having an abortion in their lifetime [36].

Yet abortion in Australia remains subject to scrutiny, restrictions, and stigma [26]. Conscientious objection on religious, moral or personal grounds is a right for pharmacists and medical providers [37], with an estimated 14% of obstetricians and gynecologists having beliefs opposed to providing abortion [38]. Some providers support only some reasons for choosing an abortion – for example, reporting they are not comfortable with abortion for “social” or economic reasons [38]. Only 7% of GPs were registered as prescribers of medication abortion in 2021, despite broad eligibility [39]. Providers are hesitant to provide abortion care, may keep their abortion provision secret, or are discriminated against for providing abortion care [40–42]. Abortion seekers, particularly in rural areas, report secrecy, stigma, and confusion about how to access care [40, 43, 44].

### Study design

This was a phenomenological qualitative study using in-depth interviews to learn about experiences of abortion care-seeking in Australia during the COVID-19 pandemic. Phenomenology is well-suited to exploring the lived experiences of individuals [45]. Through in-depth interviews, we examined individual accounts of stigma as they are shaped socially and relationally through interactions with healthcare workers in the context of macro-level abortion stigma and other structural factors influencing the health system, including the pandemic.

### Eligibility and recruitment

Inclusion criteria were being at least 18 years of age, able to participate in an interview in English, and having sought abortion care in Australia between March 2020 and November 2022, regardless of whether an abortion was ultimately obtained. Recruitment materials specified “people” rather than “women” to be inclusive of any gender identity. We recruited from December 2020 to November 2022 using social media materials (Twitter, Facebook, and Instagram) posted by sexual and reproductive health organizations in Australia. Flyers were also placed in the waiting rooms of clinics that provided high numbers of abortion services and agreed to support study recruitment. We aimed to recruit a sample of at least 20 participants with diversity in terms of state or territory where they sought an abortion, the type of procedure (medication or surgical), gestational age at time of abortion, as well as sociodemographic factors including age, rurality, and racial/ethnic background. Potential participants were directed to a Qualtrics survey with screening questions (location, age, date and type of abortion, pandemic restrictions at time of abortion). We invited eligible respondents to review consent materials and an Explanatory Statement before scheduling an interview. Recruitment ended after we conducted 20 interviews, at which point we continued to contact everyone eligible who had already submitted an expression of interest.

### Data collection and management

The principal investigator conducted the interviews, which were audio recorded using the Zoom videoconferencing platform. At the beginning of each interview, the investigator reviewed consent information verbally, answered any questions the participant shared, and audio recorded verbal consent. Interviews followed a semi-structured interview guide with questions about participants’ abortion trajectory, from finding out they were pregnant, to information-gathering, to seeking care, as well as their retrospective reflections about the experience. The participant led the discussion based on what was most important for them, with prompts and follow-up questions by the interviewer. Interviews lasted between 30 and 105 minutes, after which participants were given a AUD40 gift card in respectful recognition of their time and any inconvenience.

Recordings were automatically transcribed through Zoom. Student research assistants compared all transcripts to the audio file to correct transcription errors. All files were stored on secure Monash University platforms. Ethics approval was obtained from the Monash University Human Research Ethics Committee (Project 30926).

### Data analysis

Our analysis and interpretation were informed by Strong, Coast and Nandigiri’s micro, meso, and macro conceptualization of the socioecological model of abortion stigma [13] and by the eight domains of person-centered care for reproductive health equity defined by Sudhinaraset et al. [46], which have been applied to abortion quality of care by Altshuler and Whaley [47]: dignity, autonomy, privacy/ confidentiality, communication, social support, supportive care, trust, and health facility environment [46, 47].

SM, MB, and SW conducted a qualitative thematic analysis of the interview transcripts. Drawing on the stigma and quality of care frameworks described above and SM and SW’s familiarity with the data, we developed a preliminary coding framework to categorize the interactions between abortion seekers and healthcare workers and identify how stigma, as well as high-quality, stigma-free interactions, manifested. We included interactions with all types of healthcare workers, including medical receptionists, sonographers, general practitioners, nurses, and surgeons. The coding framework also included macro factors that influenced experiences of care, including the pandemic, rural health system limitations, and abortion laws and regulations. Using the preliminary coding framework, we each coded the same four transcripts, selected to represent a diversity of abortion experiences. We discussed any discrepancies or lack of clarity in codes, collapsed some codes, expanded others, and updated the codebook. SM and SW then coded the remaining transcripts using Dedoose data analysis software, meeting with MB periodically to discuss emerging adjustments to the codebook.

The final analysis phase was carried out by SM and MB, who have expertise in abortion stigma, quality of care, and typologies. We define typologies as interpretive categories based on the clustering of thematic codes. They are an interpretive lens created through the process of data analysis to allow comparisons and patterns to be drawn across a cohort of diverse experiences [48]. In this study, typologies refer to categories of interactions between healthcare workers and abortion seekers. We created the typologies by analytically grouping together positive and negative experiences reflecting stigma and quality of care in an iterative process. This process was conducted inductively until the sub-codes within each typology were cohesive and we could articulate a clear distinction between typologies. We did not presume that the positive and negative typologies would align. Through iterative analysis, we ended with five negative typologies which aligned with five corresponding positive typologies.

## Results

SM interviewed 24 participants ranging from 20 to 40 years of age (Table 1). All identified as cisgender women. They lived in seven of the eight Australian states and territories, and 10 resided in non-metropolitan areas. Between March 2020 and November 2022, 21 participants had one abortion, two participants had two abortions each, and one sought an abortion but did not ultimately obtain one after having a spontaneous miscarriage. This leaves 25 experiences of complete abortion in the study, of which fourteen were medication, nine surgical, and two hospital-based medical inductions. Gestational age at time of abortion ranged from five to 28 weeks.

**Table 1:** Participant (n = 24) and abortion (n = 25) characteristics

Participant characteristics	n = 24
**Age range (mean = 29.6)**	
20–24	5 (21%)
25–29	6 (25%)
30–34	9 (38%)
35+	4 (17%)
**State/Territory**	
Victoria	10 (42%)
Queensland	6 (25%)
New South Wales	3 (13%)
South Australia	2 (8%)
Australian Capital Territory	1 (4%)
Western Australia	1 (4%)
Northern Territory	1 (4%)
Tasmania	0 (0%)
**Rurality**	
Urban	14 (58%)
Regional	10 (42%)
**Race/ethnicity**	
Caucasian	20 (83%)
Australian Aboriginal	1 (4%)
Asian	1 (4%)
Hispanic	1 (4%)
Multiracial	1 (4%)
**Abortion characteristics**	**n = 25**
**Procedure type**	
Medication	14 (56%)
Surgical	9 (36%)
Hospital-based induction	2 (8%)
**Year of abortion**	
2020	5 (20%)
2021	11 (44%)
2022	9 (36%)

### Typologies

We constructed ten typologies of interactions in abortion care (Table 2): five categories of negative interactions that reflect stigmatizing, low-quality care and five categories of positive interactions that reflect non-stigmatizing, high-quality care.

**Table 2:** Positive and negative typologies of interactions between abortion seekers and healthcare workers

1. Interactions that reflect stigmatizing, low-quality abortion care	2. Interactions that reflect non-stigmatizing, high-quality abortion care
1.1 Creating barriers to abortion access	2.1 Actively helping people access abortion care
1.2 Judging, blaming, questioning, or punishing abortion seekers	2.2 Actively validating abortion decision
1.3 Interactions that do not respond to evolving emotional or information needs of the client	2.3 Interactions responsive to evolving emotional and information needs
1.4 Making assumptions about reproductive intentions and related preferences for care	2.4 Aligning abortion provision with client reproductive intentions and care preferences
1.5 Minimized interactions that compromise the quality and safety of the service	2.5 Providing holistic and high-quality care to ensure a safe service

Each negative typology demonstrates tangible experiences described by abortion seekers that are explicitly or implicitly influenced by abortion stigma within the healthcare interaction. Each positive typology aligns with a negative typology and represents the nature and type of non-stigmatizing and high-quality interactions that are possible when that form of stigma is absent. The 10 typologies are described below, including the different manifestations of each typology and illustrative quotes exemplifying the experiences of participants. Interactions that reflect stigmatizing, low-quality abortion care are shown in Table 3, and those that demonstrate non-stigmatizing, high-quality abortion care are shown in Table 4.

### Typologies of stigmatizing and low-quality abortion care

**Table 3:** Typologies of interactions that reflect stigmatizing and low-quality abortion care, with codes representing manifestations of each category

Typology	Codes (manifestations of the typology)
1.1 Creating barriers to abortion access	Gatekeeping or deliberately delaying care; insufficient information provided when booking/referring; denial of care
1.2 Judging, blaming, questioning, or punishing abortion seekers	Questioned decision; don’t do this again; woman bears responsibility; withholding or minimizing pain relief; singled out or treated differently for abortion
1.3 Interactions that do not respond to evolving emotional and information needs of the client	Insufficient attention to emotional needs during and after care; insufficient time and attention to support decisions about abortion care; pushed towards one method; unfriendly and cold
1.4 Making assumptions about reproductive intentions and related preferences for care	Ultrasound wishes not respected; pushing contraception; assume client wants to continue the pregnancy; assume client wants to have the abortion
1.5 Minimized interactions that compromise the quality and safety of the service	Insufficient aftercare information; lack of follow up; recovery rushed, pushed out the door

### 1.1 Creating barriers to abortion access

Healthcare workers behaved in a range of ways – from subtle to blatant – that created barriers to abortion access. The main behaviors were providing insufficient information to abortion seekers, delaying or gatekeeping access to the service, or denying care altogether. Several participants described hurried and uninformative interactions with their provider. Even when GPs referred onwards, brief interactions left participants confused and unsure how to obtain the service they needed. As one participant described, “I said [to the GP] […] ‘We’d like to access termination services. […] I’ve tried to look into it, but I don’t know who to access, who to talk to. Is that something that you can help me navigate?’ And he just kind of got a bit uncomfortable and just said, ‘we don’t do that here. There’s some GPs that do.’ […] I was literally on the phone with him, for I think three minutes in total. […] I left more confused I think than anything. And then they’re like, right, here’s your bill for $75” (ID18). This cursory care was also experienced in cases when the provider agreed to prescribe medication abortion.

Another form of creating barriers was delaying access, as in the case of one participant who was incorrectly informed (whether intentionally or not) that she could not obtain an abortion until later in pregnancy. “[The receptionist] asked me how far along I was. […] I thought about six weeks. And she said, well, we can’t actually do anything till nine weeks.” (ID20). Delaying care also corresponded with providers questioning abortion seekers’ decisions, as with a GP who “was kind of hesitant to give it to me, and he’s like, ‘No, I’d rather like you think about it and come back Monday’” (ID15).

Some participants described experiencing denial of care. Two participants said a receptionist denied them access to a GP because they were seeking an abortion. As one said, “[when booking] I said, ‘Oh, by the way this is for a termination of pregnancy. That’s why I need the appointment.’ And the receptionist on the phone just said ‘no, we don’t do that.’ But she was very short and very abrupt.” (ID19). Further, some participants had difficulty finding a pharmacy that would fill their prescription for medication abortion.

### 1.2 Judging, blaming, questioning, or punishing abortion seekers

Participants described direct and implicit communications from healthcare workers that made them feel blamed, judged, or punished for their pregnancy and subsequent abortion. Some participants felt their decision was questioned by providers, for example being told “you’ll regret [an abortion] if you’re 30” (ID02) or asked, “If I told you [that] you were having twins, would it change your mind?” (ID05). Further, some healthcare providers communicated that abortion should not be repeated. “[The GP said] ‘you’re not allowed to do this [abortion] again.’ […] Scolding me like I was some young girl who’d been super irresponsible, not like an adult woman who is aware of, like her decisions” (ID15).

Some participants said they felt that the responsibility for the pregnancy was placed solely on the woman. As one participant told us, “I wish there was more information about male contraception. The fact that the whole situation falls on the person with the uterus is, is really upsetting” (ID04). Other participants felt that the need for pain relief was minimized, or pain relief was withheld from them. “[If] I had adequate information, it would mean I could get better pain medication in advance rather than having to go to the emergency department in the midst of COVID” (ID08). Participants also described being treated differently for abortion, as opposed to other types of health procedures. “I think the GP should have […] had more information and less opinions […], like they would with any other procedure” (ID02).

### 1.3 Interactions that do not respond to abortion seekers’ emotional and information needs

Participants described interactions with healthcare workers that did not respond to their emotional and information needs at different points in the abortion pathway. These interactions often involved healthcare workers having an unfriendly or cold demeanor. In some cases, participants were not told about the different types of procedures available to them, preventing them from making an informed decision. “[I] would have much preferred surgical but I don’t feel like that was like adequately explained to me at the time. […] I remember seeing on my Medicare bill that the doctor had charged me for like, $180 for counselling. I don’t feel like, you know, I was counselled in any way. In some ways I’m grateful for the fact that she didn’t try to talk me out of [an abortion]. But in other ways, I sort of feel like that [appointment] should have involved an exploration of the process of a medical termination versus a surgical termination” (ID08). Others similarly said they would have liked more time with their provider to learn more about what the service would entail.

Many participants also said their providers did not attend to their emotional needs. Some abortion seekers wanted more emotional support during the appointment, while others wanted more structured parallel support like counselling services. One said, “I feel like they definitely could have done more to comfort me and just make the process a little bit easier for me. […] To have a bit more time with the doctor to explain the procedure to me […] I just feel like that would have comforted me” (ID17). Another concurred that emotional support was “really, really lacking. It was very like, very medical focused” (ID21). Further, participants often described the healthcare workers as “disconnected”, “abrupt”, “cold”, “didn’t give a crap”, or having “no sympathy”. Particularly impactful was a participant who said, “Everybody in that clinic was cold […]. It was horrible. From the moment I got there to the moment I left” (ID22).

### 1.4 Making assumptions about reproductive intentions and related preferences for care

In many interactions, healthcare workers made assumptions about participants’ reproductive intentions. Some providers incorrectly assumed that the person wanted to remain pregnant, or alternatively, that they had already decided they wanted an abortion. Providers also made assumptions about whether the participant wanted to discuss contraception or be shown the ultrasound image.

Diverse providers incorrectly assumed their client wanted to be pregnant, commonly during bloodwork, ultrasound, or when seeking a referral. As one participant said, “[the sonographer] was acting very excited for me, and asking if I wanted images saved, and asking when my due date was, and that sort of thing. Which was really really hard to deal with” (ID25). Another participant told us, “It was horrible. I faked it and I just played along because I didn’t want to have that conversation [about abortion]. That felt like taking a bullet” (ID11). In some cases, general practitioners incorrectly assumed the participant wanted to be pregnant, and sometimes failed to provide options for abortion care based on these assumptions. As one participant said, “You think you’d read the room and maybe think, the girl might need some options. […] I wouldn’t go back to that GP.” (ID09).

In a few cases, providers incorrectly assumed the client was sure about the decision to have an abortion. One participant who had been coerced to have an abortion by her partner said she would have wanted support from the provider to explore her decision. “Maybe there were assumptions made about me, that I’d been really firm on my decision. […] I wish someone had a dug a little bit deeper” (ID21).

Providers also made assumptions about client preferences for ultrasound and contraception. Multiple participants were shown the ultrasound despite expressing that they did not want to see it. Others requested to see it, but their request was refused. Multiple participants also said they felt the abortion providers overemphasized contraception. As one said, “They also were really pushing an IUD onto me, and I found that really opportunistic” (ID02). Another noted, “they are reminding [you about contraception] every time, and […] you feel that pressure, feel like you have to do it” (ID23).

### 1.5 Minimized interactions that compromise the quality and safety of abortion care

Many participants described receiving insufficient procedural or aftercare information, follow up, or time to support the recovery process. This led some to call after hours hotlines or visit the emergency department when they weren’t sure if their symptoms were dangerous. A participant said about her visit to the emergency department, “I just didn’t really feel like I should have been there, but I didn’t know how else to go about it rather than go to the GP […]. I just don’t feel like they prepared me for just how much [bleeding] it would be” (ID21). Further, several participants described being “pushed out the door” (ID17) or rushed through recovery after their surgical abortion. Several were released while still dizzy from anesthesia or without their accompanier being notified, with potential ramifications for their safety. One said, “They hadn’t called [my partner] or anything, and I was still quite, you know, drowsy, [and] stumbling in the car park. […] My partner [happened to] look up in his rear vision mirror and saw me” (ID22). Some were told about aftercare instructions while they were still drowsy, leading them to forget their medication. “I was supposed to have antibiotics that night […] Why didn’t they give [my partner] the information?” (ID22). Further, many participants said they did not receive a follow-up call or the results of their laboratory tests confirming success of the abortion. “I didn’t receive any follow-up support. […] I think that they maybe, should have offered me something, or even just a follow-up phone call” (ID17).

### Typologies of non-stigmatizing and high-quality abortion care

**Table 4:** Typologies of interactions that reflect non-stigmatizing and high-quality abortion care, with codes representing manifestations of each category

Typology	Codes (manifestations of the typology)
2.1 Actively helping people access abortion care	Proactively supporting access; took time to provide information when booking/referring
2.2 Actively validating abortion decision	No judgement; explicit validation of decision
2.3 Interactions responsive to evolving emotional and information needs	Emotional support and comfort during and beyond service; time and effort to support decisions and tailor abortion care based on preferences
2.4 Aligning abortion provision with client reproductive intentions and service preferences	Client preferences for ultrasound met and respected; contraceptive discussion aligned with client wants and needs; find out and support pregnancy preferences andintentions
2.5 Providing holistic and high-quality care to ensure a safe service	Sufficient aftercare info for safe management; proactive follow up to ensure safety and wellbeing of client; supportive interactions during recovery

### 2.1 Actively helping people access abortion care

Participants described proactive support from their providers to ensure they could access abortion care. Some providers, who did not themselves provide abortion care because of health systems constraints, proactively provided abortion information, or helped organise the referral and subsequent appointments. For example, one participant described how “[the GP] went through what the process was, and that there were no options available for me in my local area, and that she would have to refer me to somebody else. […] Yeah, she got me in for the bloods next, all straight away, and then over to the ultrasound, and then she gave me follow-up call. I think she tried to call me four times […]. And I was amazed that, you know, somebody would take so much time out of their day just to check on me, to make sure that I had […] got the referral” (ID22). Another participant described receiving assistance from a sonographer. “She was like, ‘you know you have options […]. You can go to the hospital tonight, if you want to, or I’ll call your GP and we can book you in.’ […] She made a call to my GPs office, my GP just happened to be there working late and they got me booked in […]. That was like really efficient and a relief, it meant that I came away from the appointment with a clear plan, and I think that was really important to me at the time. Like yeah again, the sense of agency and some sort of picture of what was about to happen” (ID08).

### 2.2 Actively validating abortion decision

Participants also experienced interactions that actively validated and supported their decision to have an abortion with different types of healthcare workers along the care pathway. Some providers actively verbalised their support, for example saying, “there’s no right or wrong’” (ID18), “we don’t judge anybody” (ID24), and “you have every right to be here” (ID08). One abortion seeker said, “[the doctor] just listened and she just said, this is your choice. You know what you need, you know your life, you know what you’re capable of, and what you’re doing isn’t wrong. You’re just making a decision. […] She just said all the right things” (ID13). Participants described such support as “affirming”, “empathetic”, “supportive” and “amazing”. Participants sometimes anticipated their decision being questioned when seeking care, and commented favorably when this did not happen. “[The GP] didn’t ask, like any questions that would even like imply judgment like ‘How did this happen’. […] She was just incredibly supportive and really like outcome [and] action oriented” (ID01).

### 2.3 Interactions responsive to evolving emotional and information needs

Many participants described interactions in which healthcare workers offered comfort and emotional support during and after the abortion service, supported an informed decision-making process, or ensured the service aligned with their needs and preferences. Some providers spent ample time to provide options and engaged in a dialectical process with their client to ensure their abortion care was tailored to their preferences. “[The doctor] gave me the options. We weighed them up together and then made the decision together. I sort of already knew what I wanted to go towards, but he was really good […] in talking to me about […] both options in detail” (ID25). Many of these providers went above and beyond the constraints of health systems. For example, one participant told us, “I hope everyone gets access to a GP as lovely as mine was. […] Having access to someone who will take the time to sit with you and go through all the options. Like, we were certainly extending beyond standard appointments. In my time with her we were really going through things and understanding what was going to happen” (ID01).

Participants shared a range of experiences in which their emotional needs were at the center of the interaction. Healthcare workers often comforted participants during the service, for example giving them a hug, stroking their head, and being caring and friendly. One participant who could not have a support person due to COVID-19 restrictions said the “nurse ended up actually coming into the procedure with me and staying with me until I woke up. Incredible” (ID04). These experiences are reflected in the sentiment of one participant who chose a clinic because “they treat you as a person, not a number” (ID06). Some services followed up after the abortion to check on the emotional and physical wellbeing of their client or by offering counseling services.

### 2.4 Aligning abortion provision with client reproductive intentions and service preferences

Participants described positive interactions in which the provider sought to understand their reproductive intentions and service-related preferences, and then aimed to provide care accordingly. A few participants said their provider asked them directly about their pregnancy intentions. “I felt like [the GP] was quite kind, you know, and like he asked me what was I wanting to do” (ID21). Providers then tailored the service accordingly. “[My partner and I] were trying to decide what to do, which was very scary because obviously we weren’t expecting this [pregnancy]. So, the doctor gave me some time to think. […] Gave me a timeline, if I was to terminate, what sort of termination I would have, and also gave me some vitamins if I was to go ahead with the pregnancy” (ID03).

Ultrasound and contraceptive counseling were areas in which some participants were supported to have an experience that aligned with their preferences. Some were offered a choice of whether to see the ultrasound image. “I really didn’t want [to] get an ultrasound at all […], especially by myself. […] They were like, ‘someone will come in with you. You don’t have to like, look at anything [on the screen].’ And they were just really, really good at calming all the things that I was bringing up” (ID04). Some providers let the client lead any discussion about contraceptives and provided information and support if requested by the client. As one participant told us, “I said, you know I want to have Implanon [the contraceptive implant] put in on my arm. And [we] just sort of talked through that.” (ID09). Another participant who declined an IUD at the time of the abortion appreciated the follow-up a few weeks later. “They contacted me back and we spoke about contraception […] and I said I would like to get it done” (ID24).

### 2.5 Providing holistic and high-quality care to ensure safety

Some participants described receiving sufficient aftercare information, follow up, and supportive care during recovery, which prepared them to manage their abortion safely. In some cases, they received this level of information from just one of the many people they interacted with on their pathway to care. “It was [not the doctor but] the pharmacist that went into detail and said, if this happens, then you gotta go into emergency and […] gave me the […] tools for me to make an informed decision at home, or when to escalate something if something was [going] wrong” (ID16).

Participants also described ways that healthcare workers were supportive and helpful during the recovery process after a surgical abortion. “There was a nurse like right next to me when I woke up […] I think that was very good and comforting to have someone there immediately upon waking up. And I think it was nice, they had like music playing in the recovery” (ID19). High quality care left participants with a sense that seeking an abortion was a legitimate option and they were deserving of good care. One participant said, “I woke up and then had a nice male nurse that was sort of fussing around. I think that [they had] snacks, and they sort of say, have something to eat. So that, that [I] was just feeling as though it was a normal, fine thing to be doing” (ID09).

Some providers also followed up after the procedure to ensure the safety and wellbeing of their client. One participant said, “because it was [about to be] a long weekend, they called me [on] the Friday, to make sure I was all okay” (ID18). Another said their GP called them to proactively organize follow-up care. “She’s just going to go through, probably do some blood and stuff again, just to make sure that it was successful” (ID22).

### Macro-level factors influencing abortion experiences

This section illustrates how the above-mentioned experiences of abortion stigma at the individual (micro) level and in interactions at the hospital, clinic, and provider (meso) level were influenced by macro (legal, regulatory, and health system) factors.

Gestational age limits influenced the experiences of many participants. Those nearing the 63-day gestational age limit for medication abortion had an urgent need to find timely appointments with a willing provider – a particular challenge among rural participants. One participant seeking surgical abortion at 28 weeks due to a fetal condition was subject to regulations requiring approval from multiple healthcare providers after a defined gestational age. “That really put in perspective, the significance of this medical board. Because even though we had made the decision to terminate the pregnancy, it wasn’t our decision. If that makes sense, it was someone else’s decision” (ID07).

Some participants lacked a choice in method due to limited provision in rural areas. “There was no one who did [surgical termination of pregnancy] in North Queensland. If I wanted the surgery, I was going to have to go [over 1,500 km] to Brisbane” (ID15). Others had to travel long distances to the nearest provider – sometimes to receive low quality care. In one particularly harrowing experience, the participant described waiting in an abortion clinic for hours, dressed in only a shirt and underwear, in an overcrowded waiting room. Healthcare workers were rude, rushed, uncompassionate, and showed little concern for protecting patient privacy. She said, “if there were more options […] there’d be a bit more competition. But they’re the only [abortion provider]. So, they really get to pick and choose as they want. The facilities were terrible, the support was terrible” (ID22).

COVID-19 regulations, which varied over time and by location since March 2020, are a structural factor that influenced the abortion-seeking experiences of most participants in the study. Many were subject to restrictions prohibiting an accompanying person from entering the abortion service. Further, the pandemic contributed to long wait times, overburdened healthcare workers, and appointment cancellations and delays – all of which were common experiences in this study.

These macro-level factors – from regulations to health system pressures during the pandemic and in rural areas – limited the abilities of abortion seekers and providers to ensure an optimal pathway to care.

## Discussion

This study contributes to the evidence base about the interrelationship between abortion stigma and quality of care – which is only beginning to be understood. The findings show that without addressing stigma at multiple levels, equitable access to high-quality abortion care will be difficult to achieve. This aligns with calls for a focus on stigma as part of quality improvement across a range of health services and conditions [14, 49].

### The impact of stigma on quality of care at the micro- and meso-levels

The positive typologies illustrate how a lack of stigma enables patient-centered abortion care. Abortion seekers clearly articulated the positive aspects of their healthcare interactions, which reflect the supportive care, communication, autonomy and dignity domains of the Person-Centered Care Framework for Reproductive Health Equity [46]. They discussed the importance of healthcare workers removing barriers to abortion access, validating their abortion decision, engaging empathetically, and responding to their evolving emotional needs. While the abortion process was not emotionally difficult for some participants, others felt they needed extra support through counselling services. Compassionate care has been articulated as a priority by abortion seekers in various settings [19, 47], and is essential in all contexts, not just formal counselling [25, 50]. The Inroads abortion stigma and quality of care framework emphasizes the importance of kindness and empathy in abortion care and the necessity of including this in provider training [25].

The negative typologies illustrate stigmatizing behaviors detracting from high-quality care. Participants who felt insufficiently informed, unsupported, and rushed through care explicitly described how they wished their experience had been different. In contrast, those who experienced a dialectical decision-making process described the benefits of having adequate time to ask questions and discuss options based on comprehensive information. Both the Australian Commission’s Quality Framework for Health Care [51] and the International Planned Parenthood Federation’s quality of care framework for sexual and reproductive healthcare [52] emphasize shared decision-making about care. The latter recommends that providers develop strong interpersonal skills and highlights the need to listen patiently to clients’ needs and concerns, answer questions, and use clear, non-judgmental and supportive language [52].

Pain management for medication abortion was an area in which abortion seekers alluded to the intersection between quality of care and stigma. The few participants with high levels of pain said they felt unprepared in terms of pain relief, and several sought after-hours care or went to the emergency department. Two participants said they suspected their pain medication was withheld by a GP or pharmacist because they were having an abortion – perhaps as a form of punishment. This aligns with the Inroads abortion stigma and quality of care framework [25], which identifies the withholding of “appropriate abortion or pain management technologies” as a manifestation of stigma. Pain is the most common side effect in medication abortion, with severe pain experienced by a substantial minority of those taking it [53]. As such, the risk that stigma could negatively impact pain management practices is consequential for many abortion seekers.

Our results also illustrate the ways in which healthcare worker assumptions about reproductive intentions can lead to stigmatizing interactions, reducing quality of care. This is typified by encounters, commonly during ultrasound and bloodwork, in which healthcare workers congratulated abortion seekers on their pregnancy. Participants expressed a desire for tailored care based on an understanding of their needs and preferences, particularly when choosing an abortion method and during contraceptive counselling. In line with the ‘acceptability/patient-centered’ dimension of the Inroads abortion stigma and quality of care framework [25], all types of healthcare worker interacting with abortion seekers – not just clinicians – could benefit from building skills for non-judgmental communication that avoids assumptions.

This study also demonstrates how stigma (or a lack thereof) in interactions with providers can enhance or limit abortion seekers’ autonomy. We found that some providers did not ascertain the ultrasound viewing preferences of abortion seekers, and in a few cases ignored the client’s explicit request to see or not see the image. A study in Norway similarly identified ultrasound before abortion as a source of “autonomy under pressure” [54]. Further, some participants in our study said they felt pressured to adopt contraception, resonating with findings from Scotland that abortion seekers may feel coerced to take up contraception [55]. A study at a US-based abortion clinic found that autonomy is more important to women in decisions about contraception that it is in decisions about general healthcare [56]. In light of concerns globally that contraceptive uptake is not always genuinely voluntary [57], it is important to conceptualize how stigma may limit autonomy in the provision of the range of services surrounding abortion care, including ultrasound and contraception.

### The impact of macro-level factors on interactions and experiences

In this study, three main categories of macro-level factors influenced and shaped experiences in care. First, regulatory and health system abortion stigma, such as the health system’s relegation of abortion from public to private service-delivery models [58] and laws regulating gestational age limits and their exemptions [59]. Second, the systemic staffing and resource limitations of rural health services resulting in a lack of options for pregnant people in rural and remote areas [32, 60, 61]. Third, the coronavirus pandemic – a backdrop to all experiences in this study – exacerbated health system challenges and barriers to abortion care in Australia and beyond [26].

Our findings illustrate how macro-level abortion stigma and other factors at the health system level interact with the stigmatizing beliefs and behaviors of local healthcare workers to hinder access to high-quality abortion care. A first example is a participant (ID20) who received incorrect information from a receptionist that would have delayed her care beyond nine weeks gestational age; this would have eliminated the option for medication abortion due to regulations across Australia. A second example is a participant with a diagnosed fetal condition (ID07) who needed medical board approval after 24 weeks gestational age. She was ultimately able to obtain an abortion but felt the decision to do so was not truly her own, particularly as she was told that one member of the board did not approve the request. A final example is the many participants who encountered an overburdened health system, leading to unsatisfactory interactions. These interactions reflected a range of factors including insufficient time in health appointments and the limited abortion provider and referral options in rural areas, and were undoubtedly exacerbated by the pandemic’s impact on the health system. Importantly, our study also identified ways in which healthcare workers proactively helped abortion seekers overcome the meso- and macro-level stigma they encountered – for example by seeking out unlisted abortion prescribers, calling clinics to book time-sensitive appointments, and providing multiple referral options at different distances and costs.

Drawing on these examples, we argue that the negative interactions demonstrated in this study reflect the interrelationship between stigmatizing beliefs among healthcare workers, macro-level abortion stigma in the policy and regulatory environment, and other structural health system limitations exacerbated during the pandemic, particular in rural areas.

### Improving quality of care by addressing abortion stigma

Our findings demonstrate a need to reduce stigmatizing interactions on pathways to abortion care. Recent systematic and scoping reviews have identified a need for theory-based and tested stigma reduction interventions for health services [2, 14], particularly in sexual and reproductive health [49, 62]. Because stigma exacerbates intersectional barriers to healthcare [12, 49], stigma-reduction efforts will contribute to equitable access, and is of relevance to other maternal and reproductive health services where clients may be disrespected, stigmatized, mistreated, and their preferences not considered [62, 63].

Our results, based on the experiences and perspectives of Australian abortion seekers, provide empirical evidence that can inform training and quality improvement. Stigmatizing interactions commonly occurred with receptionists, in ultrasound, during bloodwork, and with referring GPs, but were also experienced in dedicated abortion clinics. These findings demonstrate the importance of ongoing training for all types of healthcare workers and staff, as emphasized in the Inroads abortion stigma and quality of care framework [25]. The negative typologies developed in this study provide case studies of subtle to overt forms of abortion stigma in healthcare interactions. This can help providers identify blind spots in their own practice and consider bystander approaches if they observe these behaviors among colleagues. Drawing on the positive typologies, training can also be designed to help healthcare workers develop proactive strategies to overcome common stigma-related barriers such as conscientious objection and gatekeeping [37]. The positive typologies can be used to connect the negative manifestations of stigma directly with facets of person-centered care [46], and generate positive emotions, which can help generate behavior change [64, 65]. Finally, the findings can inform the development of values clarification approaches focused on the harms of stigma in healthcare interactions. Values clarification is a training approach that has been used to change abortion-related knowledge and attitudes in healthcare workers by encouraging examination of personal morals and values [49, 62, 66].

There is growing recognition that stigma-reduction efforts must focus beyond the individual level to be effective [5]. Yet recent reviews have found a lack of interventions focused on the structural and societal drivers of stigma [49, 62, 67]. While training necessarily engages individual healthcare workers, there are ways to also integrate a meso- and macro- focus. First, whole-of-institution approaches can support change at multiple levels, for example by sensitizing management, institutionalizing de-stigmatization protocols and policies, and shifting institutional culture around abortion. Whole-school approaches to violence prevention have shown promising results using multi-level interventions [68, 69] and may serve as an exemplar. Second, provider training can move beyond the individual by focusing on positionality and power differentials between abortion consumers and healthcare workers [5]. Third, training can incorporate discussions of ‘abortion exceptionalism’ – “the idea that abortion is regulated both differently and more stringently than other medical procedures that are comparable to abortion in complexity and safety” [70], to illustrate the legal and regulatory structures that reinforce stigma.

### Strengths and limitations

This study considers interactions between abortion seekers and health care workers and the structural factors shaping them. It is complex, if not impossible, to disentangle to what extent negative client experiences reflect stigma, health system limitations, the poor bedside manner of an individual provider, or a combination of these. Drawing on ample evidence that stigma intersects with and exacerbates barriers to care and inhibits quality of care [9], we did not seek to fully differentiate between stigma and other health system limitations, but rather explore them in their interrelatedness. Future research can build on this early conceptualization of the influence of macro-level abortion stigma on micro and meso experiences in care.

A strength of this analysis is the centering of participant voices in developing typologies about stigmatizing and non-stigmatizing care. Study participants described the pandemic as one of many influences on their experiences, but overwhelming emphasized interpersonal interactions and other preexisting health system limitations as the main factors limiting quality of care; these are the focus of this paper. We acknowledge that some findings about poor experiences of care may have been heightened by the influence of the pandemic on the health system. Implications of this study include the need to prepare for future pandemics and other disasters by proactively addressing the systemic challenges limiting equitable access to abortion care, which are likely to be exacerbated in disaster conditions.

A challenge of the analysis approach was creating discrete typologies when the manifestations of stigma are interrelated. Some forms of stigma raised in the interviews (internalized, anticipated, and how these relate to low expectations of care) were not examined in this analysis as they were beyond the defined focus on interactions in healthcare.

Our choice of qualitative methods provided rich empirical data from the perspective of abortion seekers with a range of experiences in terms of rurality, type of abortion, and gestational age at time of abortion. However, the sample was predominantly white and entirely cis-gender, Victorian participants were overrepresented, and we did not recruit anyone who tried to obtain an abortion but failed to access it. This limits our analysis of intersectional experiences of abortion stigma. Further, participants self-selected into the study, and the sample may reflect those with particularly strong positive or negative experiences or with strong opinions about abortion. Challenges recruiting diverse samples remain in health research [71], and stigma research and intervention development should continue to prioritize integrating an expanding range of perspectives.

## Conclusions

Stigma cannot be ignored when considering quality improvement in abortion care. The typologies presented in this paper can inform efforts to reduce stigma in healthcare interactions during abortion care, particularly in ultrasound, bloodwork, contraceptive counseling, and when being referred. Abortion stigma in the health system and at the regulatory level, as well as structural factors limiting health system functioning generally, also shape the behavior of healthcare workers – and in doing so, contribute to poor experiences in care. These structural factors too must be addressed in stigma-reduction strategies. Approaches that identify and intervene on stigmatizing interactions between healthcare consumers and providers will have broad applicability to other maternal and reproductive health services globally.

## Data Availability

The datasets generated and/or analyzed during the current study are not publicly available for privacy reasons but are available from the corresponding author on reasonable request.
